# Exploring gastric bacterial community in young pigs

**DOI:** 10.1371/journal.pone.0173029

**Published:** 2017-03-01

**Authors:** Vincenzo Motta, Paolo Trevisi, Francesca Bertolini, Anisa Ribani, Giuseppina Schiavo, Luca Fontanesi, Paolo Bosi

**Affiliations:** Department of Agricultural and Food Sciences (DISTAL), University of Bologna, Bologna, Italy; Wageningen University, NETHERLANDS

## Abstract

Microbiota plays an important role in the homeostasis of the gastrointestinal tract. Understanding the variations of the commensal microbiota composition is crucial for a more efficient control of enteric infectious diseases and for the reduction of the use of antibiotics in animal production, which are the main points of interest for improved animal healthcare and welfare and for consumer health protection. Even though the intestinal microbiota has been extensively studied, little is known about the gastric microbiota. This pilot study was aimed at a descriptive analysis of the gastric microbiota in healthy pigs and at the identification of any differences among four potentially distinct microbial niches in the stomach. Gastric mucosal samples from the oxyntic area, the pylorus and the gastric groove, and a sample of gastric contents were collected from four healthy weaned pigs. Bacterial DNA was isolated and extracted from each sample and amplicons from the V6 region of the 16S rRNA gene were sequenced using Ion Torrent PGM. The data were analysed by an “unsupervised” and a “supervised” approach in the Ribosomal Database Project (RDP) pipeline. Proteobacteria was the dominant phylum in all the samples. Differences in bacterial community composition were found between mucosal and content samples (one-way ANOSIM pairwise post hoc test, *p* < 0.05); instead, the different mucosal regions did not show differences between them. The mucosal samples were characterised by *Herbiconiux* and *Brevundimonas*, two genera which include cellulolytic and xylanolytic strains. Nevertheless, additional larger trials are needed to support the data presented in this pilot study and to increase the knowledge regarding the resident microbiota of the stomach.

## Introduction

The importance of the microbiota to the health status of the gastrointestinal tract is widely recognised. Over the years, the Microbial Ecology of the GI tract has been extensively explored [1] but the stomach ecosystem has received less attention; this was due to the technical limitations and to the fact that the gastric environment was considered too inhospitable. The potentiality of the gastric environment as a microbial niche was reconsidered after the discovery of *Helicobacter pylori* and thanks to successive technological advances [2]. In recent years, one of the most used methods for exploring the microbial diversity of an environment is 16S rRNA profiling conducted using Next Generation Sequencing (NGS) approaches; however, the studies which use these techniques to analyse the stomach microbiota of monogastric animals (non-human) are still infrequent [3,4]. In particular, as regards the pig, only the work [4] of Mann et al. (2014) which also analyses the gastric microbiota using this technique was found.

The stomach is a system of temporary storage and pre-processing of the food bolus for additional digestion and absorption; this system is directed by an integrated control (neural, hormonal, paracrine) which takes into account the different signals (chemicals, nutrients, xenobiota components) from the luminal content [5]. In the stomach three anatomic parts (fundus, corpus and antrum) and two functional regions -oxyntic (acid secretion) and pyloric (gastrin secretion) glandular mucosa- can be distinguished.

This anatomical and functional geography within the stomach has been investigated in several ways [6,7].

In the pig, the oxyntic glands are found in the cardia gland and the fundic gland regions (OXY), while the antral-type mucous glands are found in the pyloric gland region (PYL).

Furthermore, in the pig stomach, regional differences were also observed in the protective layer of the mucus[8,9] which represents the first line of interaction between bacteria and the gastrointestinal tract [10].

The question arises as to whether the different gastric regions may represent distinct niches for diverse communities within that ecosystem; this possibility has been investigated for monogastric mammals in only a few studies, such as in humans (corpus and antrum) [11] and in horses (squamous, glandular, antral) [3] but the identification of a specific gastric microbiota, excluding *H*. *pylori*, requaires additional investigation [12].

The present study fits into the context of our other studies regarding the pig gastric mucosa [7,13–15] our aim was to contribute to the description of the gastric microbiota, in particular, of the pig, and to identify possible differences of the bacterial community in different parts of the stomach.

For this purpose, Next generation semiconductor-based sequencing of the V6 hypervariable region of the 16S rRNA gene was used on gastric mucosal samples from the oxyntic area (acid production), the pylorus (gastrin secretion)and the gastric groove, a point in the small curvature close to cardia, (immunological function) [13] and also from the gastric content.

## Materials and methods

### Pigs and sample collection

The procedures were conducted in compliance with Italian laws regarding experimental animals and were approved by the Ethic-Scientific Committee for Experiments on Animals of the University of Bologna. Four crossbred (Large White *x* Landrace) healthy weaned pigs (6.5 weeks of age, 15.30 kg average body weight), normally fed a standard post-weaning diet (ingredient composition: corn 38.2%, barley13%, wheat middlings 16%, soybean meal, 50% crude protein 13%, dried milk whey 9%, potato protein concentrate 4%, soybean oil 3%, vitamin-mineral premix 1%, dicalcium phosphate 1.2%, calcium carbonate 0.61%, salt 0.3%, L-lysine HCl 0.38%, Dl-methionine 0.11%, L-threonine 0.15%, L-tryptophan 0.05%), were anaesthetised 1 h after the morning meal with sodium thiopental (10 mg/kg body weight) and were then slaughtered by intracardiac injection (Tanax, 0.5 mL/kg body weight; Intervet Italia, Peschiera Borromeo, Italy). For each subject, the stomach was removed, and gastric mucosal samples from the oxyntic area, the pylorus, and the small curvature close to cardia (hereinafter called Groove) were collected. A sample of gastric contents was also collected from each pig; in total 16 samples were obtained (4 from each pig). The samplings in each pig and each stomach region were carried out using sterile instruments to avoid potential cross-contamination of the microbial DNA.

The samples were stored at -80°C until use. The bacterial DNA was isolated and extracted using QiaAmp DNA Stool Mini Kit (Qiagen, Hilden, Germany). The protocol followed the manufacturer’s instructions with a pretreatment step with TES buffer + Lysozyme at 37°C for two hours. After isolation, the purified DNA was eluted in 50 μl of elution buffer. The quality and purity of the isolated DNA was checked using spectrophotometry on the NanoDrop (Fisher Scientific, Schwerte, Germany).

### Library generation and sequencing

Polymerase chain reaction (PCR) amplification of the V6 hypervariable region from the 16S rRNA gene was carried out with a pool of 5 forward primers and 4 reverse primers pooled equimolar as described by Huber et al. [16]. Phusion Hot Start Flex 2X Master Mix (New England Biolabs Inc., Beverly, MA) was used following the manufacturer's protocol for a 25μl reaction; the PCR conditions were as follows: 98°C for 30 s, followed by 35 cycles at 98°C for 5 s, 61°C for 8 s, 72°C for 12 s, and a final elongation step at 72°C for 5 s.

Ion Torrent sequencing was obtained from 16 different DNA libraries. The libraries were constructed using the aforementioned amplified products, after ExoSAP-IT (USB Corporation, Cleveland, Ohio, USA) treatment, and 16 equimolar pools of amplicons were obtained. The preparation of the libraries was carried out according to the instructions for the Ion Torrent Personal Genome Machine (PGM; Life Technologies,*Carlsbad*, *CA*) sequencing of short amplicons; for each library, 200 ng of amplified DNA was end-repaired and ligated with a specific barcode, in total 16 different barcodes were used, using the Ion Xpress™ Barcode Adapters 1–16 kits (Life Technologies). Subsequently, the protocol included the following steps: quantification of each library with the Ion Library Quantitation Kit (Life Technologies) by quantitative polymerase chain reaction (qPCR), utilising a StepOnePlus™ Real-Time PCR System (Life Technologies); equimolar pooling of the 16 barcoded libraries, amplification by emulsion PCR with the Ion One Touch^TM^ 200 Template kit (Life Technologies), and purification and sequencing with the Ion PGM^TM^ Sequencing 200 kit using a Ion 314 chip (Life Technologies), following the manufacturer's protocols.

Raw reads of all the samples were deposited at the EBI Short Read Archive (SRA) under the study accession number ERP010584.

### Data analysis

A total of 353,656 Raw reads from sequencing were filtered for length ≥ 70 and average quality ≥ 20. The following steps were carried out in the Ribosomal Database Project (RDP) pipeline of the RDP release 11.3, using both ‘unsupervised’ and ‘supervised’ methods (http://rdp.cme.msu.edu/) [17]; primer matching and trimming were performed by the ‘Pipeline Initial Processor’ of the RDP pipeline (Maximum number of Ns: 0; Max forward primer distance: 0; Max reverse primer distance: 0; Minimum sequence length: 50), chimera checking was carried out using the tool ‘Find Chimeras’ in DECIPHER (Database Enabled Code for Ideal Probe Hybridization Employing R) [18]. Non-chimeric sequences were aligned by RDP Aligner and the sequence reads not covering the V6 region were eliminated. After quality control steps, 86,731 total sequences were obtained.

For bacterial taxonomy assignment, the RDP-classifier (version 2.2) [19] was used with 50% as confidence value threshold and gene copy number adjustment. Operational Taxonomic Unit (OTU) analysis was carried out on a clustering at the 97% identity threshold using the complete linkage clustering algorithm.

For the unsupervised approach 2961 reads (the lowest number of reads recovered in a single sample) were randomly subsampled from each sample (package GUniFrac in R) in order to minimise the impact of the varied sequencing depth among samples. For the supervised approach, sequence tag data were normalised to relative abundance within the sample for analysis and visualisation. The statistical software PAST version 2.17 [20] was used to analyse the abundance data of the reads assigned to taxa within the samples, with one-way ANOSIM (Analysis of Similarities) testing significance of the difference between the groups of samples based on differences in the gastric region. Cluster Analysis and Principal Components Analysis were used to generate graphical representations of the differences in community composition. The SIMPER (Similarity Percentage) analysis was used to identify the specific genera with the greatest contribution to the differences observed between the groups identified.

## Results

### Unsupervised approach

After rarefaction, 2,020 OUTs were represented among the 47,376 reads from the16 samples in this study. The sequencing depth and the total OTU richness within individual samples are reported in Table 1 and the per sample rarefaction curves are reported in S1 Fig.

**Table 1:** Distribution of reads and Operational Taxonomic Units (OTUs), before (pre rarefaction) and after (post rarefaction) normalization by subsampling to the lowest number of reads recovered in a single sample.

Individual	Sample	pre rarefaction		post rarefaction	
		Reads	OTUs	Reads	OTUs
1	Content	9273	813	2961	476
2	Content	7504	573	2961	366
3	Content	5967	562	2961	401
4	Content	9408	787	2961	450
1	Oxyntic	5660	401	2961	311
2	Oxyntic	4648	319	2961	264
3	Oxyntic	4891	396	2961	335
4	Oxyntic	6096	460	2961	351
1	Pyloric	4033	428	2961	381
2	Pyloric	2961	332	2961	332
3	Pyloric	4127	391	2961	335
4	Pyloric	4412	392	2961	338
1	Groove	3586	383	2961	356
2	Groove	4025	374	2961	337
3	Groove	4717	428	2961	365
4	Groove	5423	457	2961	370

**Fig 1:**
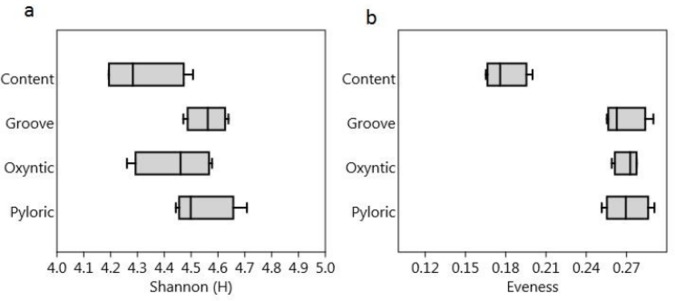
Box-plot of the Shannon-Weaver (a) and Eveness (b) index values in the samples from different regions of the stomach.

The Shannon-Weaver diversity index and Eveness were calculated for each sample and the average indices at each of the points (Content, Groove, Oxyntic and Pyloric) showed a quite similar diversity, though with a lower equitability (eveness) of the OTUs within the Content samples (Fig 1).

**Fig 2:**
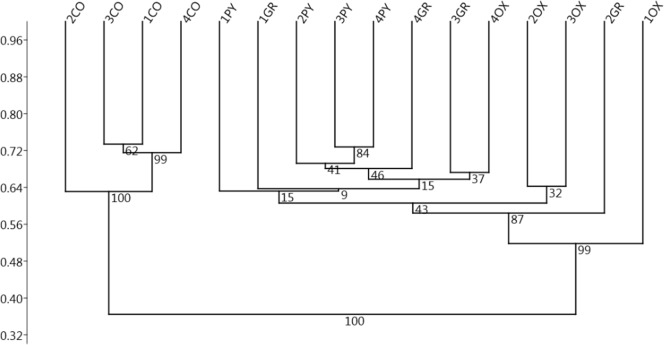
Unweighted Pair Group Method with Arithmetic Mean (UPGMA) Cluster analysis (Bray-Curtis distance) of the samples of the gastric mucosa based on the abundance of the OTUs. Labels indicate the pig identification number and the stomach region (CO: contents, OX: oxyntic, PY: pyloric, GR: groove) with 10,000 Bootstrap resamplings used: the bootstrap Probability values (BP) are shown at the nodes.

The Cluster analysis based on the abundance of the different OTUs in the samples (Fig 2) showed two well-supported clusters; one consisted of all the samples from the content and the other consisted of all the samples from the mucosa. The clustering did not show an individual effect or an effect of the regions in the mucosa; however, 3 out of the 4 samples from pyloric region formed a sub-cluster poorly supported by bootstrap probability (BP = 41).

The null hypothesis, that there were no significant differences in community structure based on the sample type (Content, Oxyntic, Pyloric, Groove), was rejected by one-way ANOSIM with an R of 0.632 (*p* = 0.0001). The pairwise post hoc test showed significant differences (*p* < 0.05) between the mucosal and the content samples, but no significant differences were seen for the comparisons between the different mucosal regions (Table 2).

**Table 2:** ANOSIM (analysis of similarities) post hoc test based on abundance of OTUs in samples.

	Content	Oxyntic	Pyloric	Groove
Content		0.029	0.033	0.028
Oxyntic	0.029		0.059	0.088
Pyloric	0.033	0.059		0.715
Groove	0.028	0.088	0.715	

Significant differences in pairwise comparisons are highlighted in gray. The one-way ANOSIM was performed on Bray-Curtis distance with 10,000 permutations, the samples were grouped according to the point of origin.

### Supervised approach

The quality checked reads were analysed using the taxonomy-supervised method in the RDP pipeline, which consists of a ‘taxonomy binning’ of the reads on the basis of the existing bacterial taxonomy. This approach has some advantages, such as the least computational effort required, minor sensibility at the sequencing errors and easier handling of the data [21].

**Fig 3:**
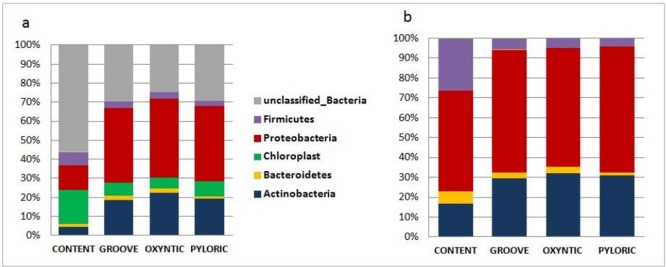
Relative abundance (average) of the principal phyla in the different stomach regions. Before and after removal of unclassified Bacteria and Chloroplast reads. Verrucomicrobia, Fusobacteria and Acidobacteria phyla (not visible in the Figure) were also found with abundances <1%.

First of all the classification at the phylum level showed the dominance of the unclassified bacterial reads and chloroplast sequences, which represented chloroplast from the ingested vegetal matter, in the content samples and a uniform distribution of the phyla in the different regions of the mucosa (Fig 3). In order to focus the study on the classifiable bacterial community, the unclassified and plasmidial sequences were excluded from additional analysis.

The dominant phylum was Proteobacteria with 50% (on average) in the content samples and 60.9% (on average) in the mucosal samples, the second phylum was Firmicutes for the content samples (27.5% on average) and Actinobacteria for mucosal samples (30.8% on average) followed by Bacteroidetes (5.53% in the content samples; 2.78% in the mucosal samples). One-way ANOSIM showed weak (R = 0.272) but significant (*p* = 0.0075) differences in community structure; in the pairwise post hoc test, significant differences (*p* < 0.05) were reported between the mucosal and the content samples but not for the comparisons between the different mucosal regions.

**Fig 4:**
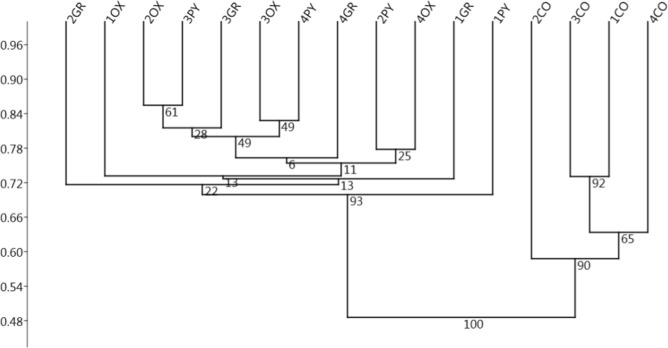
Unweighted Pair Group Method with Arithmetic Mean (UPGMA) Cluster analysis (Bray-Curtis distance) on the samples of the gastric mucosa based on the relative abundance of the sequence reads classified at the genus level. Labels indicate the pig identification number and the stomach region (CO: contents, OX: oxyntic, PY: pyloric, GR: groove) with 10,000 Bootstrap resamplings used; bootstrap probability values (BP) are shown at nodes.

The classification at the genus level identified 238 genera in total. The Cluster analysis, based on the relative abundance of the Genera (Fig 4), showed a situation similar to that seen in the unsupervised approach with a cluster for all samples from the content samples and another cluster for all the mucosal samples. No clusterings by subject or for different mucosal regions were found.

One-way ANOSIM again showed significant differences (R = 0.354; *p* = 0.0018) between the mucosal and the content samples but not for the comparisons between the different mucosal regions (Table 3).

**Table 3:** ANOSIM post hoc test based on relative abundance of genera in samples.

	Content	Oxyntic	Pyloric	Groove
Content		0.031	0.027	0.031
Oxyntic	0.031		0.969	0.441
Pyloric	0.027	0.969		0.623
Groove	0.031	0.441	0.623	

Significant differences in pairwise comparisons are highlighted in gray. The one-way ANOSIM was performed on Bray-Curtis distance with 10,000 permutations the samples were grouped according to the point of origin.

The SIMPER analysis (Table 4) was carried out in order to identify the Genera wich most influenced the difference between the bacterial communities of mucosa and content. The overall average dissimilarity (Mucosa VS Content) was 51.43%. The SIMPER analysis showed that the differences between the bacterial communities of the gastric mucosa are primarily driven by the dominance of the genus *Herbiconiux* with an average abundance of 41.9% in the mucosal samples and 21.6% in the content samples. Other principal genera characterising the mucosa are *Brevundimonas* and *Moritella;* instead, *Pasteurella*, *Streptococcus*, *Lactobacillus* and *Lactococcus* characterise the contents.

**Table 4:** Similarity Percentage (SIMPER) genera contribution.

Genus	Contribution	Mean ab. Cont.	Mean ab. Muc.
Herbiconiux	10.15	21.60	41.90
Pasteurella	3.45	7.51	0.62
Streptococcus	3.09	6.79	0.64
Brevundimonas	2.77	8.29	12.90
Moritella	2.41	5.01	9.83
Lactobacillus	2.30	5.58	0.98
Lactococcus	1.96	4.23	0.32
Phenylobacterium	1.34	2.55	0.53
Ochrobactrum	1.19	3.31	4.62
Prevotella	1.18	2.74	0.42
Delftia	1.10	1.76	3.85
Stenotrophomonas	0.98	1.02	2.74
Curtobacterium	0.68	1.41	0.05
Pseudomonas	0.59	1.38	0.21
Cloacibacterium	0.55	1.01	0.17
Eikenella	0.55	1.17	0.15

“Contribution” represents the average contribution of a given Genus to the average dissimilarity between samples (overall mean = 51.43%). “Mean ab. Cont.” is the average relative abundance (in %) in content samples “Mean ab. Muc.” is the average relative abundance (in %) in mucosal samples. The list of genera is not exhaustive, an arbitrary threshold of a mean contribution of 0.5 was used as a cut-off.

**Fig 5:**
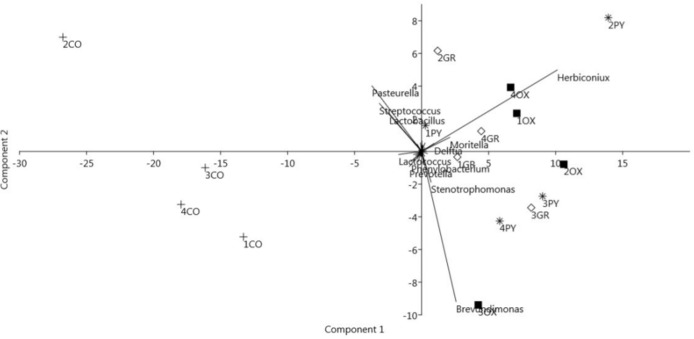
Biplot of the Principal Component Analysis of the stomach samples based on the relative abundance of bacterial genera per sample. Labels indicate the pig identification number and the stomach region (+ CO: contents, ■ OX: oxyntic, * PY: pyloric, ◊ GR: groove). Names of the bacterial genera with the highest loadings are plotted as vectors according to their correlation to the first two components.

Principal component analysis (PCA) regarding the relative abundances of the bacterial genera showed that the 78.5% of variance in the data could be explained by the first two principal components. This analysis confirmed the subdivision of the samples into two main clusters along the first component (Fig 5), a cluster consisting of contents samples and a cluster containing samples obtained from the mucosa and the PCA biplot (Fig 5) shows the influence on this clustering of genera also reported by SIMPER analysis.

## Discussion

The current study revealed values of alpha diversity of the pig gastric microbiota similar in the different areas of the stomach anlysed (Oxyntic, Pyloric, Gastric Groove and Content), and comparable to those reported by Mann (2014). The bacterial community composition, analysed at different taxonomic levels (Phyla, Genera, OTUs), did not show differences in the distribution of the bacterial populations between the different areas of the gastric mucosa. This indicated that the anatomic and physiological differences in the different gastric areas (see Introduction) may not directly impact the bacterial community, which is probably more influenced by the outer mucus layer. Unfortunately, however, the exact location of the microbiota in the stomach of mammals is still unknown [22].

Instead, significant differences in bacterial distribution at the phyla, genera and OTU levels are observed between the mucosa and gastric content. Even if our results should be treated with caution since they are based on a limited number of replicates, this differentiation between the mucosal and the luminal microbiota has already been described for the gastrointestinal tract of other mammals [23], indicating that the digesta bacterial community may not be sufficiently informative of the bacterial community associated with the gastric mucosa.

First of all regarding the taxonomic classification of the reads, the large number of unclassifiable bacterial sequences, approximately 55% in the content and 30% in the mucosa at the phylum level is first of all evident. This gives a general idea regarding the bacterial diversity still unexplored. When focusing on the reads taxonomically identified, it can be seen that the principal phyla (Proteobacteria, Actinobacteria, Firmicutes and Bacteroidetes) are those usually reported for the gastric ecosystem of mammals [22]. In our study, however, the dominant phylum was Proteobacteria, in contrast to what was reported by Mann et al. (2014) for the non-glandular area of the pig stomach in which Firmicutes represented the dominant phylum with *Lactobacillus* genus. This difference could be due to the different gastric area analysed; in fact, the non-glandular stomach area is reported as that which hosts primarily lactobacilli [24,25].

In the present study, classification at the genus level identified *Pasteurella*, *Streptococcus* and *Lactobacillus* as those most characterising the gastric content. In the pig, *Pasteurella* is mainly reported for the upper respiratory tract [26] and it is linked to diseases of the respiratory system [27]. Streptococci and lactobacilli, for which our study found relative abundances slightly in favour of streptococci (6.79% vs. 5.58%), have already been described in the pig stomach as competitors in the post weaning period [28].

Instead, the bacterial genera which characterise the gastric mucosa were *Herbiconiux* and *Brevundimonas*; *Herbiconiux* included strains associated with plant matter [29,30] which has never been described before in the gastrointestinal tract of mammals. The genus *Brevundimonas* includes opportunistic strains ubiquitous in the environment, and a strain with growth requirements typical of *Helicobacter* wich has been reported in the gastric mucosa of dogs [31]. The presence of both *Herbiconiux* and *Brevundimonas* was also observed in a set of samples of the pyloric mucosa analysed, using the same technique, in another our study (unpublished data), but with lower abundances (about 4%), this could be explained by the association of these bacteria with the last meal ingested. Interestingly, *Herbiconiux* and *Brevundimonas* include strains which can degrade cellulose and xylan found in the gut of some insects [32,33] and it was noted that the bacteria associated with the plant matter of the meal could represent an inoculum of functionally similar strains in mammals [34]. Furthermore, the action of cellulase and xylanase enzymes has already been the subject of interest in pig feeding studies [6,35]; the possibility of exogenous xylanase activity in the pig gastric environment has also been reported [36]. Mann et al. (2014), for example, suggest for *Prevotella* the degrading activity of hemicelluloses in the stomach of pig; regrettably, microbiota profiling through the 16s rRNA gene cannot provide information regarding the functionality and vitality of the bacteria reported.

Finally, must not be forgotten that the bacterial community of the stomach is more directly influenced by the last meal ingested [37] and by the sampling and the management of the animals [3], and that the scarcity of studies in the literature explicitly addressing the gastric microbiota makes the formulation of stronger hypotheses regarding the impact of different gastric locations on microbiota composition difficult. The present results indicated that proper designs could be formulated for the additional identification and isolation of variables which modify gastric microbiota in the pig; nevertheless, the exploratory nature of this pilot study must be pointed out, and larger studies focused on the stomach would provide validation of the data presented herein. It is hoped, therefore, that the efforts now dedicated to the description of the gut microbiota will stimulate additional studies involving the gastric ecosystem.

## Supporting information

S1 Fig Per Sample Rarefaction Curves. Numbers indicate the pig identification number and the initials indicate stomach region (CO, contents; OX, oxyntic; PY, pyloric; GR, groove).
(PDF)
