# Supplementary material for: Exploring gastric bacterial community in young pigs
Source: PLoS One. 2017 Mar 1;12(3):e0173029. doi: 10.1371/journal.pone.0173029 (PMC5332105; doi:10.1371/journal.pone.0173029)

Figure S1 Per Sample Rarefaction Curves. Numbers indicate the pig identification number and the initials indicate stomach region (CO, contents; OX, oxyntic; PY, pyloric; GR, groove).

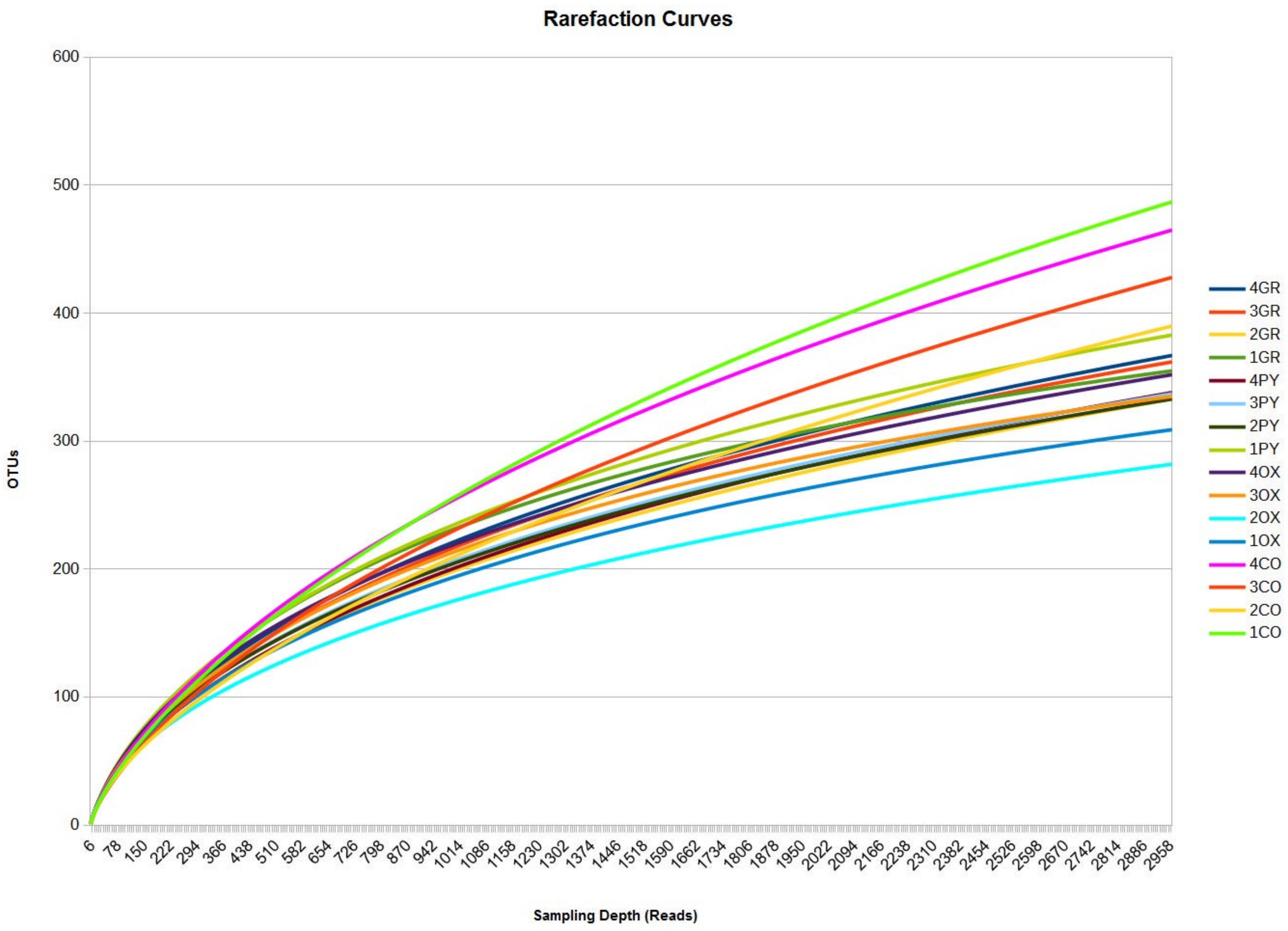

Supplement: S1 Fig — Numbers indicate the pig identification number and the initials indicate stomach region (CO, contents; OX, oxyntic; PY, pyloric; GR, groove). (PDF) [file pone.0173029.s001.pdf]
